# Environmental Enrichment Prevents Methamphetamine-Induced Spatial Memory Deficits and Obsessive-Compulsive Behavior in Rats

**Published:** 2017-01

**Authors:** Samira Hajheidari, Hossein Miladi-Gorji, Imanollah Bigdeli

**Affiliations:** 1 Faculty of Psychology and Educational Sciences, University of Semnan, Semnan, Iran.; 2 Laboratory of Animal Addiction Models, Research Center and Department of Physiology, School of Medicine, Semnan University of Medical Sciences, Semnan, Iran.; 3 Faculty of Educational Sciences and Psychology, Ferdowsi University of Mashhad, Mashhad, Iran.

**Keywords:** *Environmental Enrichment*, *Grooming*, *Methamphetamine*, *Obsessive-Compulsive Disorder*, *Spatial Learning and Memory*

## Abstract

**Objective: **This study was designed to examine the effect of environmental enrichment during methamphetamine (METH) dependency and withdrawal on methamphetamine-induced spatial learning and memory deficits and obsessive-compulsive behavior.

**Method:** Adult male Wistar rats (200 ± 10 g) chronically received bi-daily doses of METH (2 mg/kg, sc, with 12 hours intervals) for 14 days. Rats reared in standard (SE) or enriched environment (EE) during the development of dependence on METH and withdrawal. Then, they were tested for spatial learning and memory (the water maze), and obsessive-compulsive behavior as grooming behavior in METH-withdrawn rats.

**Results:** The results revealed that the Sal/EE and METH/EE rats reared in EE spent more time in the target zone on the water maze and displayed significantly increased proximity to the platform compared to their control groups. METH withdrawn rats reared in EE displayed less grooming behavior than METH/SE group.

**Conclusion**: Our findings revealed EE ameliorates METH-induced spatial memory deficits and obsessive-compulsive behavior in rats.

Chronic exposure to METH may produce long-term changes in the brain structure, function and synaptic plasticity (1), apoptosis (2), neurodegeneration (3) and neurotoxicity (4) in the hippocampus, these changes can affect aspects of behavior, learning and memory, and structures important for spatial learning and memory that enables an animal to recognize its position in the charted world. In this regard, it has been shown that chronic METH administration is associated with neurocognitive impairment (2), impairment of spatial working memory (5), and short- and long-term retention of a novel object recognition task (6). Our previous findings indicated that induction of methamphetamine-induced sensitization impaired spatial memory 30 and 120 minutes after injection, which persisted even after 30 days of withdrawal, but spared spatial working memory (7).

Chronic administration of METH also produces changes in mesolimbic, nigrostriatal systems and pre-frontal cortex, which then causes the rewarding effects and craving of drug (8, 9), obsessive-compulsive disorder (OCD) as stereotypy behavior (10, 11), and locomotor activity (12). Grooming behavior is an innate behavior in animals similar to obsessive–compulsive disorder (OCD) in humans (13), which is higher after chronic administration of METH (10, 11).

A recent study suggested that addictive behavior occurs following cognitive-affective mechanisms triggered by drug-related environmental cues, which in turn activate reinstatement of compulsive drug-seeking behavior (14). Thus, the reversal or prevention of METH-induced behavioral and cognitive disorders could be a useful method for the treatment of relapse after prolonged abstinence.

In our previous studies, environmental enrichment (EE) for 30 days during spontaneous METH withdrawal reduced the voluntary consumption of METH and also anxiety and depressive-like behaviors in rats.

In EE models, laboratory animals are placed in large cages with physical stimuli, including small toys and running wheel, which are much richer than the standard housing, and allow animals to explore, play and exercise; in this condition, the animal could have more control over the environment (15).

In another study in our laboratory, rats exposed to an enriched environment during induction of METH dependence showed greater decrease in behavioral withdrawal symptoms (16). In addition, the environmental enrichment could prevent spatial learning deficits induced by aging and chronic stress (17, 18), and enhance neurogenesis in the hippocampus, which is associated with improved spatial memory (19, 20). Thus, a more important question would be whether EE could blunt the deleterious effects of chronic administration of METH during METH dependence and withdrawal. Therefore, the aim of this study was to investigate whether exposure to an EE during induction of METH dependence and spontaneous withdrawal would attenuate METH-induced spatial learning and memory deficits, and OCD behavior in METH-withdrawn rats.

## Materials and Methods

***Animals, Methamphetamine Administration and Housing Conditions***

Male Wistar rats (200 ± 10 g) (n = 62) were housed at a 12-h light/dark cycle at 22–24◦C temperature, with food and water ad libitum. The Methamphetamine hydrochloride (Sigma–Aldrich, M 8750) was dissolved in 0.9% saline, and the rats were chronically treated with twice-daily subcutaneous injections of METH with a dose of 2 mg/kg (sc) at 12 hours intervals, for 14 days, as described previously (15). Control rats were similarly injected with saline. Rats were placed in their home cages (standard or enriched environment) over injection period or the spontaneous withdrawal of METH. Enriched rats were placed in a large cage (96 cm × 49 cm × 38 cm) with plastic tunnels, rope, swing, balls, ramp, ladder, shelters, step, cube and a running wheel. The toys were changed every two days to maintain novelty, and control rats were placed in standard laboratory cages (42 × 34 × 15 cm) (15). 

***Morris Water Maze***

A detailed description of the maze and the tracking system has been given in our previous studies (7, 21). All rats were trained in spatial learning (two trials per day for five consecutive days). The rats were allowed to stay on the platform for 20 seconds during the intertrial interval. The escape latency (platform search time) was recorded for each trial. An average of two trials was evaluated each day. A spatial probe test was performed 24 hours after the last acquisition session, without platform. The rats were allowed to swim for 60 seconds, the time spent in a zone around the platform (20 cm radius) in each quadrant, and the proximity (the average distance from the center of the platform during the probe test) and velocity of each animal were recorded. Data were automatically collected, using a computerized video image motion analyzer (Ethovision, Noldus Information Technology).

***OCD-Like Behavior***

Rats were individually placed in a Plexiglas box (41 cm length × 33 cm height × 41 cm width) (22), and grooming behavior as OCD-like behavior was evaluated. Components of grooming behavior included vibration, face and head washing, body grooming, scratching, paw licking, head shaking and genital grooming. Grooming behavior was scored every 15 seconds for 30 minutes. Thus, the maximum of the available score during the observation period was 120 (23).

***Statically Analysis***

The data were presented as the mean ± standard error of the mean (S.E.M.). These data were analyzed, using two-way, or three- way analyses of variance (ANOVA), with repeated measures as required followed by the Tukey’s test. Statistical differences were considered significant at P<0.05.

***Experimental Protocol***

**Experiment 1**

**Figure1:**
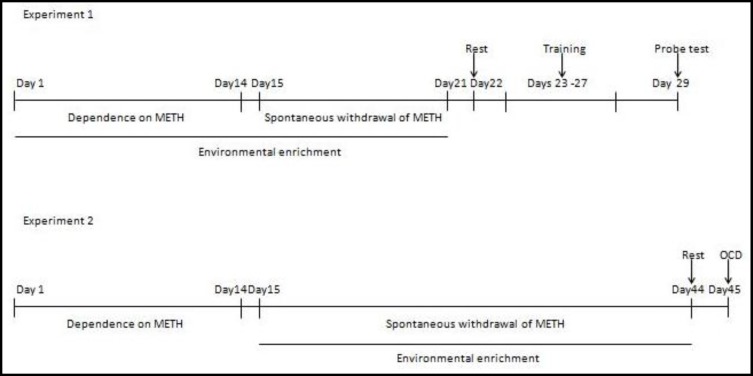
Timelines of Experiments (See Materials and Methods for Details )

This experiment examined the effects of EE on the learning and memory deficits of METH-withdrawn rats. Native rats were divided into the four following groups (n = 7-8 per group): Saline-standard environment (Sal/SE), saline-enriched environment (Sal/EE), METH-standard environment (METH/SE), and METH -enriched environment (METH/EE). The EE groups were allowed to freely exercise, play, explore over their environment during the development of dependence on METH (14 days) and during METH withdrawal (7 days). On day 22 (after a 7-day withdrawal period), all the rats were rested in standard cages and also allowed to swim for three minutes in the pool containing no platform for habituation, as described previously (24). From day 23 to 27, all rats were trained in spatial learning. A spatial probe test was performed 48 hours after the last acquisition session, without platform (day 29) (Figure 1).

**Experiment 2**

This experiment examined the effect of EE on the grooming behavior in METH-withdrawn rats. Thirty-two naive rats were divided into the following four groups (n = 8 rats per group): Saline-standard environment (Sal/SE), saline-enriched environment (Sal/EE), METH -standard environment (METH/SE) and METH-enriched environment (METH/EE). Saline or morphine was injected for 14 days for each four group. The EE groups were allowed to freely exercise, play, explore over their environment for 30 days during METH spontaneous withdrawal. On day 44, all four groups of rats were rested in standard cages. Grooming behavior (OCD) was evaluated on day 45 (Figure 1).

## Results

***Spatial Learning***

**Figure2:**
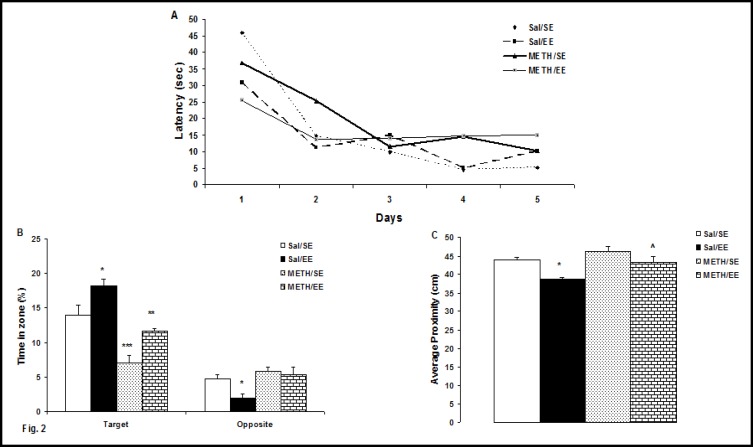
Effect of Environmental Enrichment on learning Acquisition and Memory Retention in METH-Withdrawn Rats by the WM Task

The acquisition data during the five days of training in the water maze (WM) are illustrated in Figure 2A. Two-way analyses of variance (ANOVA), with repeated measures were used to analyze the escape latencies during training. All groups learned to locate the platform during five days of training, as indicated by decreasing escape latencies as training progressed (F4, 100 = 21.7, P = 0.0001). There was no significant effects of group (F3, 25 = 1.1, P = 0.42) and no significant interaction between days and group (F12, 100 = 2, P = 0.1). In other words, there was no significant difference between groups over five days of training. 

Data related to the distance swam to reach the platform followed the same pattern as the latency. All groups traveled shorter distances to reach the platform as training progressed (F4, 100= 35.89, P = 0.0001). There was no significant difference among groups (F3, 25 = 1.65, P = 0.203), and no interaction was observed between factors (group × day) (F12, 100 = 1.97, P = 0.09). (Figure is not shown).

***Spatial Memory***

A three-way ANOVA with zones was performed with the fixed factors treatment (saline and METH) and housing condition (SE and EE) and zones (target and opposite) (Figure 2B).

Analysis revealed a significant effects of housing (F1, 50 = 4.83, P = 0.033), treatment (F1, 50 = 13.83, P = 0.001), zones (F1, 50 = 176.76, P = 0.0001), and a significant interaction between treatment and housing and zones (F4, 50=19.75, P=0.0001). The between group comparisons indicated that the Sal/EE and METH/EE groups spent significantly more time in target zone compared to the standard environment groups (P = 0.045 and P = 0.009, respectively). The MEH/SE group spent significantly less time in the target zone than Sal/SE group (P = 0.0001). In addition, the Sal/EE group spent less time in the opposite zone than Sal/SE group (P = 0.05). 

Figure 2C represents the average proximity to the platform. A two- way ANOVA revealed a significant effects of housing (F1, 25= 26.75, P = 0.0001) and treatment (F1, 25 = 28.34, P = 0.0001). Pair-wise Bonfer-roni comparisons (adjusted alpha = 0.05) revealed that average distance of the Sal/EE and METH/EE rats from the center of the platform during the probe test was significantly lower compared to the Sal/SE and METH/SE rats (P = 0.001 and P = 0.031, respectively). In other words, the Sal/EE and METH/EE rats had a significantly higher average proximity.

To control for differences in WM performance, we recorded each animal’s swimming speed, but we found no difference (F3, 25 = 2.36, P<0.095) in the swimming speeds of the four groups: Sal/SE (24.76cm/s), Sal/EE (23.03 cm/s), D/SE (22.04 cm/s), and D/EE (23.31 cm /s).

***OCD-Like Behavior***

**Figure3:**
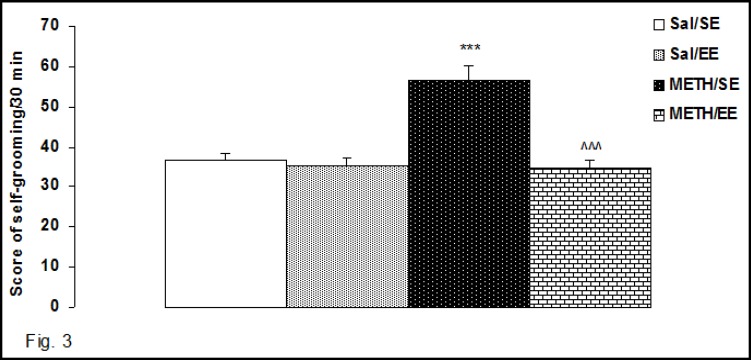
Effect of Environmental Enrichment on the Self-Grooming Signs in METH-Withdrawn Rats. The Self-Grooming Sign was lower in METH/EE Group than METH/SE.

The results of the self-grooming behaviors are shown in Figure 3. Two-way ANOVA revealed a significant effect of housing (F1, 28 = 27.17, P = 0.0001), and treatment (F1, 28 = 16.4, P = 0.002), and significant interaction between both factors (F1, 28 = 17.23, P = 0.0001) in self-grooming score. Comparisons between groups revealed that the score of self-grooming in EE METH withdrawn rats was lower than the SE METH withdrawn rats (P<0.0001). In addition, the score of self-grooming behaviors in METH/SE group was higher than Sal/SE group (P<0.0001).

All groups learned to locate the platform during the five days of training, as indicated by decreasing escape latencies as training progressed (A). The mean time spent within the 20 cm zones in the target and opposite quadrants (B). The average proximity to the platform(C). EE rats spent significantly more time in the target quadrant and had higher proximity than their SE control groups. The Sal/EE groups spent significantly less time in the opposite zone than Sal/SE group.

*P=0.05 vs Sal/SE, ***P=0.0001 vs Sal/SE, **P=0.009 vs METH/SE, ^P=0.031 vs METH/SE.

***P <0.0001 vs Sal/SE, ^^^P< 0.0001 vs METH/SE.

## Discussion

This study showed that exposure to enriched environment can prevent the methamphetamine-induced spatial memory deficits. In our previous study (16), we found no significant difference in behavioral withdrawal symptoms between the METH/SE and METH/EE groups when assessed five days after abstinence. Thus, spatial cognitive deficits observed in the METH withdrawn rats are not related to direct drug withdrawal at the start of training and testing on spatial tasks. Our results are in agreement with previous studies that showed EE was able to reduce the learning and memory deficits induced by stress and aging (17, 18, 25, 26). Our previous findings indicated that methamphetamine-induced sensitization impaired spatial memory 30 and 120 minutes after injection (7). In line with our previous study (7), we found that rats did not show impairment of learning. 

Thus, this study revealed that exposure to enriched environment during the development of dependence on METH attenuated the damaging effect of the drug. Presently, it is not clear how enriched environment during the development of dependence on METH and 7-day withdrawal period can reduce memory deficits of METH-withdrawn rats. However, it has been shown that exposure to enriched environment increased the hippocampal neurogenesis (19, 20), dopamine transporter protein in the nucleus accumbens (27) and also facilitated spatial learning and memory through glutamate AMPA receptor mediation (28) and the hippocampal astroglial pathological changes in Alzheimer's disease (29). 

Additionally, our results have shown that 30 days of exposure to enriched environment during METH spontaneous withdrawal can reduce OCD-like behavior. This finding is further supported by previous studies showing that enriched environment improved motor deficits associated with Parkinson’s and Huntington diseases (30, 31), and reduced locomotor effects of methylphenidate (32) and stereotyped behavior in deer mice (33). Presently, it is not clear how EE can reduce OCD-like behavior as the self-grooming behaviors after a 30-day cessation period. Previous findings indicate that exposure to enriched environment enhanced serotonin concentrations in the prefrontal cortex (34), decreased activation of cortical-basal ganglia circuitry (35), and increased metabolic activity in the cortex and striatum (33), which are involved in obsessive-compulsive disorders. 

## Limitations

Thus, a more important question would be whether environmental enrichment could blunt the cognitive deficits of METH even after prolonged abstinence of drug. This question cannot be answered by our present findings and requires a different experimental protocol. Also, one of the limitation of our study was the lack of the neurobiological mechanisms that should be considered in future studies. ‎

## Conclusion

Our study provided new evidence that exposure to enriched environment could improve recovery of spatial memory impairment in METH-withdrawn rats. It also diminished OCD-like behaviors in METH-withdrawn rats. These beneficial effects of enriched environment might have a potential clinical importance in spatial cognitive and locomotor deficits associated with methamphetamine use.
